# Association of has_circ_0001944 upregulations with prognosis and cancer progression in patients with colorectal cancer

**DOI:** 10.1007/s12672-022-00485-2

**Published:** 2022-04-09

**Authors:** He Duan, Jian Qiu

**Affiliations:** Department of General Surgery, The Fourth Affiliated Hospital of China Medical University, No. 4 Chongshan East Road in Huanggu District, Shenyang, 110036 Liaoning China

**Keywords:** Colorectal cancer, has_circ_0001944, miR-548b-3p, Overall survival, Recurrence-free survival

## Abstract

**Background:**

CircRNAs are functional in cancer-related processes and are promising candidates for cancer prognostic biomarkers. The study aimed to evaluate the functional and clinical significance of has_circ_0001944 in colorectal cancer (CRC), including predictive value for overall survival (OS) and recurrence-free survival (RFS), and its effect on cell growth and metastasis.

**Methods:**

This retrospective study included 133 patients with CRC. The expression of has_circ_0001944 in tissues and cells was quantified by real-time quantitative reverse transcription PCR. Receiver operating characteristics and Kaplan–Meier survival analysis were used to assess the significance of has_circ_0001944 as a prognostic marker, and its reliability was validated using multivariate regression analysis. Subsequently, XTT, transwell migration, and modified-transwell invasion assays were used to determine the behavior of the CRC cells in response to has_circ_0001944 inhibition.

**Results:**

Results of the qRT-PCR showed upregulation of has_circ_0001944 in the CRC samples compared to the normal samples. High has_circ_0001944 expression indicated shorter OS and RFS, comes down to poor prognosis. Multivariate regression analysis showed that elevated has_circ_0001944 increased the risk of death or recurrence and is a valuable prognostic factor. Following the has_circ_0001944 inhibition, the proliferation, migration and invasion of the CRC cells were reduced. miR-548b-3p was target miRNA of has_circ_0001944.

**Conclusion:**

Up-regulation of has_circ_0001944 is associated with a poor prognosis of CRC. has_circ_0001944 downregulation can slow the progression of CRC partly by targeting miR-548b-3p.

**Supplementary Information:**

The online version contains supplementary material available at 10.1007/s12672-022-00485-2.

## Introduction

Colorectal cancer (CRC), originating from the non-cancerous proliferation of mucosal epithelial cells, is one of the common malignant tumors in the digestive system [1]. According to the latest global cancer data statistics, CRC ranks third in the global cancer incidence and mortality [2]. The data collected by the National Central Cancer Registry of China shows that the rising burden of colorectal cancer is significant with an upward trend in both incidence and mortality [3]. In the past 30 years, with the low detection rate of early cancer and the absence of standard treatment strategies in some regions of China, the mortality rate of CRC has been up to 8.0% [4]. The westernized lifestyle and age structure would aggravate the occurrence of CRC and even death in the next few years. The 5-year survival rate of CRC is related to the TNM stage. The 5-year relative survival rates for stage I and II CRC are above 50%, while the rate for stage IV was about 12–13% [5]. What’s more, compared with the East Asian neighbors, China has relatively lower survival rates of CRC (less than 36%) [6]. Taking into consideration the burden of CRC, it is mandatory to ameliorate the prognosis of CRC patients. It seems a pressing demand of the day to find a potent biomarker to increase CRC prognostic prediction.

CircRNAs are closed circular non-coding RNAs that are ubiquitously present in eukaryotic cells [7]. Considering their covalently closed-loop structure, lack of free ends, and resistance to exonuclease and RNase R digestion, circRNAs are evolutionarily conserved and more stable than linear RNAs [8]. CircRNAs are abundantly expressed across numerous human cell types, providing a novel and valuable genetic type to investigate physiology and pathology in human disease [9]. Due to their circular nature resulting in the absence of a 3' overhang, circRNAs can be preserved under RNase R treatment [10]. This brings exceptional stability for circRNAs. In addition, the expression of circRNA has been demonstrated to be cell-type-specific and stage-specific [11]. Discriminative expression profiles of circRNA between normal non-malignant and cancerous cells of colorectum have supported the postulation of circRNAs dysregulation anticipated in CRC and the correlation with CRC pathology [12, 13]. The remarkable characteristics of circRNAs have provided tremendous potential for their use as biomarkers with prognostic value. Concerning the underly mechanism, the capability to act as microRNA (miRNA) sponge was one of the mechanisms of circRNAs action [14]. Hsa_circ_0001944, a stable and cytoplasmic circRNA [15], has been screened as an upregulated circRNA in CRC [16]. It functioned as a competing endogenous RNA to promote the growth and metastasis in bladder cancer cells via sponging miR-548 [17]. But the continued research about its function and clinical significance in CRC has not been gone on in existing research.

The present study collected CRC tissues and cell lines to determine the expression level of hsa_circ_0001944. Then used a cutoff value from receiver operating characteristic (ROC), stratification between the high-expression group and the low-expression group was archived and Kaplan–Meier curves were established to evaluate the association of hsa_circ_0001944 with overall survival (OS) and recurrence-free survival (RFS) in CRC patients. The cell experiments were used to determine the behavior of the CRC cells in response to has_circ_0001944 inhibition.

## Patients and methods

### Patients, samples and follow-up

This retrospective study recruited medical information and tissue samples (both cancerous and corresponding non-neoplastic tissues) from 133 confirmed histological-CRC patients who underwent enterotomy at The Fourth Affiliated Hospital of China Medical University between January 2012 and January 2015. The ethics committee of The Fourth Affiliated Hospital of China Medical University has approved this study, and all patients have signed the written formed consent about samples for scientific research. None of the patients had previously received any treatment related to cancer. Other exclusion criteria were rare and complex types of tumors including hereditary nonpolyposis colon cancer and familial adenomas. The pivotal clinicopathological data were retrieved and extracted from institutional medical records, electronic medical records systems or doctors’ notes. The Chinese Protocol of Diagnosis and Treatment of Colorectal Cancer from Chinese Society of Clinical Oncology, which is based on the AJCC/UICC, was used for the CRC clinical tumor-node-metastasis (cTNM) staging classification and pathological evaluation (histological type and histological grading) [18]. All CRC patients were followed up for tumor recurrence and survival status for up to 5 years at regular intervals. OS refers to the period of surgery to the date of death or the final point of follow-up. RFS refers to the time quantum from the date of surgery to the date of recurrent neoplasm or death.

### Cell culture

The human CRC cell lines HCT 116, LoVo, SW1116, and SW620 were acquired from Shanghai Cell bank of Chinese Academy of Sciences (Shanghai, China), apart from the human colon epithelial cell line FHC which were from Kunming Cell bank of Chinese Academy of Sciences (Kunming, China). The cells were cultured in the respective recommended media and culture conditions: McCOY's 5A (SIGMA, USA) + 10% fetal bovine serum (FBS, GIBCO, USA) and 5% CO_2_ for HCT 116; Ham's F-12K Medium (Invitrogen, USA) + 10% FBS and 5% CO_2_ for LoVo; Leibovitz's L-15 Medium (GIBCO, USA) + 10% FBS (GIBCO, USA) and 100% air for SW1116, and SW620; RPMI-1640 (GIBCO, USA) + 10% FBS (GIBCO, USA) for FHC. The culture temperature was 37 ℃.

### In vitro transfection experiment

For a transient transfection with the aim to reduce the hsa_circ_0001944 or miR-548b-3p expression, LoVo and SW620 cells were transfected with the siRNA specifically targeting hsa_circ_0001944 (si-circ_0001944), miR-548b-3p inhibitor (anti-miR-miR-548b-3p) or their negative control (si-ctr and anti-ctr), from wcgene biotech (Shanghai, Chian) using the Lipofectamine 2000 (Invitrogen, USA). The transfected cells were incubated under their respective growth conditions and the measurement of RNA expression after hsa_circ_0001944 inhibition was conducted by real-time quantitative reverse transcription PCR (qRT-PCR) after 48 h.

### Assessment of RNA expression

The total RNA from cells and the homogenate of tissue samples was isolated using Invitrogen TRIzol (USA). The circRNA was enrichened using RNase R (Geneseed, China). hsa_circ_0001944 and miR-548b-3p expression levels were evaluated using up to 1 mg of total RNA, first subjected to reverse transcription using the PrimeScript RT Master Mix (Takara, China) and then quantification using Bulge-Loop miRNA qRT-PCR Starter Kit (RiboBio, Guangzhou, China) and the responding primers specific for hsa_circ_0001944 and miR-548b-3p and their respective housekeeping gene GAPDH and U6 on a LightCycler LC480 II (Roche Diagnostics, USA). The fold changes of hsa_circ_0001944 and miR-548b-3p expression were calculated using 2^−ΔΔCt^ method.

### Cell proliferation

The cell proliferation was quantified using the Cell Proliferation Kit II (XTT) (Roche, USA). Briefly, transfected LoVo and SW620 cells were seeded at a concentration of 5 × 10^3^ cells/well in 100 µL culture medium into 96-well microplate (Corning Life Sciences, Massachusetts, USA). The cell cultures were incubated for indicated periods (0, 24, 48, and 72 h) at 37 °C. At each time point, 50 µL XTT was added per well and the microplate was incubated for another 4 h at 37 °C. The spectrophotometrical absorbance was measured using a Spectra Max M2e (Molecular Devices, USA) at 492 and 690 nm. The cell proliferation was reflected by (A_492nm_–A_690nm_).

### Cell apoptosis

The cell apoptosis was detected by Annexin V-FITC/PI Apoptosis Detection Kit (Vazyme Biotech, Jiangsu, China). Briefly, transfected LoVo or SW620 cells (3 × 10^5^) were collected and washed. 100 μL of 1 × Binding Buffer was added into the cells, and gently made to be a single cell suspension. 5 μL Annexin V-FITC and 5 μL PI Staining Solution were added into the cell suspension and mixed gently. The incubation was conducted in the dark at room temperaturefor 10 min; After the addition of 400 μL 1 × Binding Buffer, the samples were detected by flow cytometry within 1 h.

### Cell transwell assay

Transwell assay and modified transwell assay with Matrigel-coated upper inserts were used to evaluate the cell migration and invasion. A total of 4 × 10^5^ transfected LoVo and SW620 cells in serum-free medium were added to the top chamber of a non-Matrigel or Matrigel-coated 24-well Transwell chamber (8.0-μm pore membranes, Corning, USA). At the bottom chamber, a medium with 10% FBS was added. Migrated or invaded cells in the bottom chamber were stained by crystal violet after 24 h of culture. The number of migrated cells was counted using using a Leica DMI6000B epifluorescence system (Leica, USA) at five random microscopic fields.

### Target miRNA screen and biotinylated RNA pull-down assay

Bioinformatics analysis of the predicted target of hsa_circ_0001944 was performed in Interactome (https://circinteractome.nia.nih.gov/). miR-548b-3p, a downregulated and proliferation-suppressive miRNA, was one of hsa_circ_0001944 targets [19]. The 3'-biotinylated miR-548b-3p and hsa_circ_0001944 probes were designed and synthesized by Cloud-Seq (Shanghai, China). To pull down hsa_circ_0001944 by miR-548b-3p, LoVo and SW620 cells were transfected with biotinylated miR-548b-3p (biotin- miR-548b-3p) or control miRNA (biotin-ctr). After 48 h, the cells were washed and lysed to lysates following incubation with Dynabeads^™^ M-280 Streptavidin magnetic beads (Invitrogen, USA) at 4 °C for 1.5 h. The bound RNAs were purified by TRIzol for qRT-PCR analysis.

### Statistical analysis

Differences in the level of change in RNA expression between pairs of tissues were tested for significance using a paired t-test. Statistics of significance between two groups were carried out using student t-tests. One way analysis of variance (ANOVA) was employed for comparison of three or more groups. Chi-squared test was used to gauge the association between hsa_circ_0001944 levels and clinico-pathological parameters. The cutoff value for analyzing predictions of OS and RFS was obtained by the establishment of ROC curves. The Kaplan–Meier method combined with the log-rank test was employed to compare the survival curves of high-hsa_circ_0001944 and low-hsa_circ_0001944 individual groups. Multivariate Cox proportional hazards models including age, gender, cTNM, lymphovascular invasion, histopathological type and grade were used to filter the parameters that were statistically significant for OS and RFS. The level of statistical significance was set at *P* < 0.05 (all two-tailed).

## Results

### Expression of hsa_circ_0001944 in the CRC tissues was upregulated and correlated with poor prognosis

**Fig. 1:**
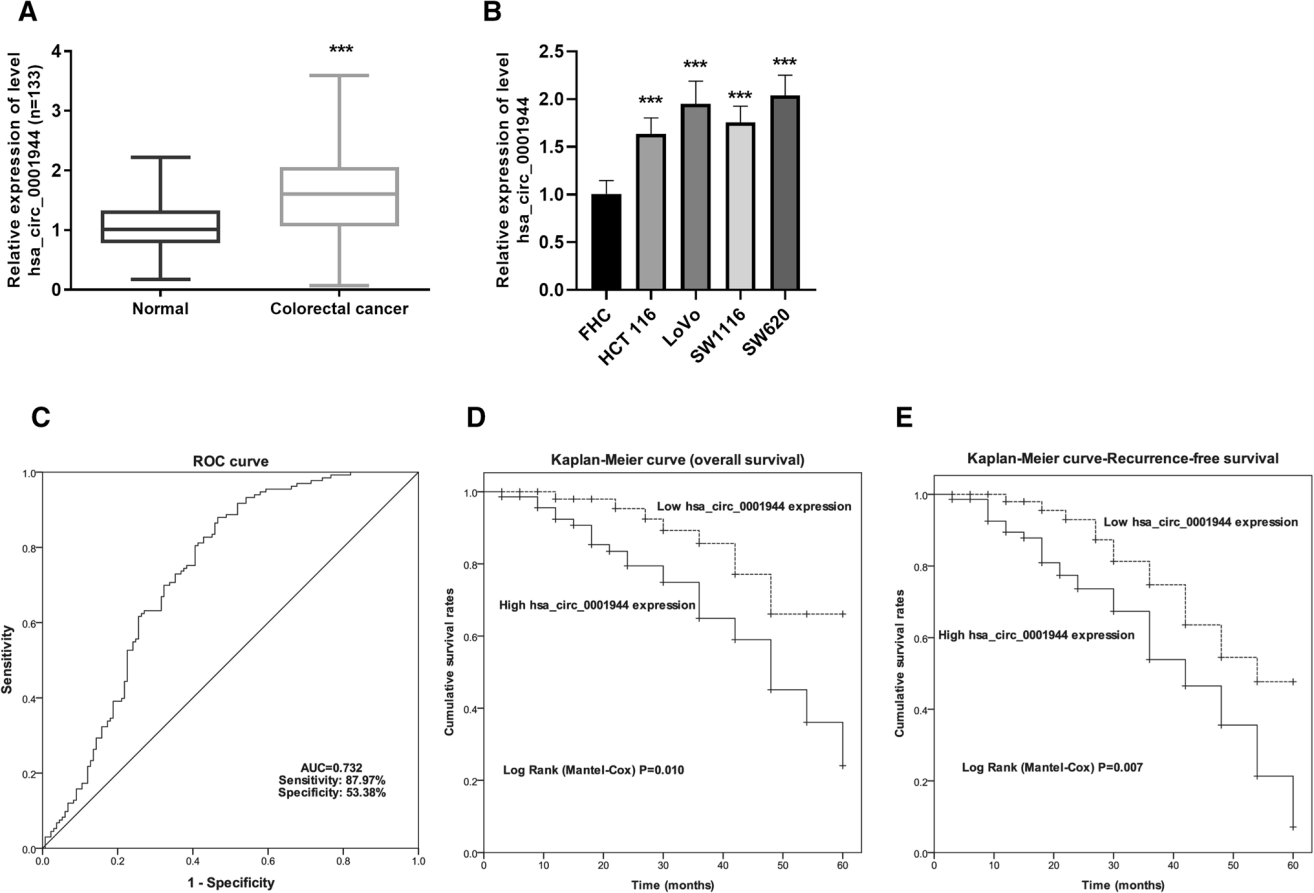
A significant increase of hsa_circ_0001944 was discovered in CRC and related to CRC poor prognosis. **A** Quantification of relative hsa_circ_0001944 in tissues (n = 133) normalized to GAPDH. Paired t test was applied. ***P < 0.001. **B** Quantification of relative hsa_circ_0001944 in cells normalized to GAPDH. One way analysis of variance was applied. Error bars are mean ± standard error from five replicate samples. ***P < 0.001. **C** ROC curve for cutoff value of hsa_circ_0001944 for survival. **D** OS was estimated using the Kaplan–Meier method, with the difference between high- and low-hsa_circ_0001944 groups compared by log-rank test. (*P* = 0.010). **E** RFS was estimated using the Kaplan–Meier method, with the difference between high- and low-hsa_circ_0001944 groups compared by log-rank test. (*P* = 0.007). *CRC* colorectal cancer, *OS* overall survival, *RFS* recurrence-free survival

The study included 133 CRC patients and the patients’ baseline data are summarized in Table 1. To investigate the expression of hsa_circ_0001944 in these pairs of samples, qRT-PCR was used. The levels of hsa_circ_0001944 in CRC tissues appear to be much greater than those in non-cancerous tissues (Fig. 1A). Consistent with this identity, the screen of a panel of human CRC cell lines (HCT 116, LoVo, SW1116, and SW620) showed higher levels of hsa_circ_0001944 than the non-malignant human colon epithelial cell line (FHC) (Fig. 1B). These results showed that hsa_circ_0001944 was highly expressed in CRC.

**Table 1:** Correlations between hsa_circ_0001944 expression and clinico-pathological parameters

Parameters	Low hsa_circ_0001944 (n = 62)	High hsa_circ_0001944 (n = 71)	*P*
Age (years)			
≤ 60	26 (41.9%)	33 (46.5%)	0.599
> 60	36 (58.1%)	38 (53.5%)	
Gender			
Female	22 (35.5%)	31 (43.7%)	0.337
Male	40 (64.5%)	40 (56.3%)	
Primary lesion			
Colon	48 (77.4%)	48 (67.6%)	0.208
Rectum	14 (22.6%)	23 (32.4%)	
cTNM stage			
I–II	40 (64.5%)	33 (46.5%)	0.037*
III–IV	22 (35.5%)	38 (53.5%)	
Lymphovascular invasion			
Absence	60 (96.8%)	50 (70.4)	0.000***
Presence	2 (3.2%)	21 (29.6)	
Histopathological type			
Tubular adenocarcinoma	57 (91.9%)	62 (87.3%)	0.387
Other	5 (8.1%)	9 (12.7%)	
Histopathological grade			
Good and moderate	48 (77.4%)	46 (64.8%)	0.110
Poor	14 (22.6%)	25 (35.2%)	

^*^P < 0.05, ***P < 0.001

To uncover the potential associations between altered hsa_circ_0001944 expression and clinical prognosis in CRC, the expression data were subjected to the establishment of ROC curve (Fig. 1C). A value of 1.575 with a maximum predictive value in ROC curve analysis was defined as the cutoff of stratification between the high-expression group (n = 62) and the low-expression group (n = 71). Concerning the parameters of CRC patients, the cTNM stage and lymphovascular invasion status showed significant differences between high and low has_circ_0001944 groups (*P* = 0.037 and P < 0.001, respectively in Table 1). From the results of Kaplan–Meier survival analysis, patients with higher levels of has_circ_0001944 expression had significantly worse OS (*P* = 0.010; Fig. 1D). In addition, the higher levels of hsa_circ_0001944 expression were found to be associated with the RFS (*P* = 0.007; Fig. 1E). The results of the Cox proportional hazard regression analyses for prognostic indicators are shown in Table 2. As anticipated, increased hsa_circ_0001944 expression was a potent prognostic marker for OS in CRC patients [HR 3.721; 95% confidence interval (CI) 1.529–9.054; *P* = 0.004] and an independent prognostic marker for RFS in CRC (HR 2.966; 95% CI 1.494–5.891; *P* = 0.002). Taken together these results suggest that hsa_circ_0001944 level is a predictive factor for OS and DFS in CRC.

**Table 2:** Multivariate analysis of clinicopathological parameters for the prognosis of overall survival and recurrence-free survival in patients with colorectal cancer

Parameters	Overall survival			Recurrence-free survival		
	HR	95% CI	*P*	HR	95%CI	*P*
Age (> 60 vs. ≤ 60)	1.869	0.666–5.242	0.235	1.031	0.414–2.568	0.948
Gender (male vs. female)	1.322	0.434–4.030	0.623	1.430	0.543–3.764	0.469
Primary lesion (rectum vs. colon)	2.348	1.020–5.407	0.045	2.139	1.062–4.305	0.033
cTNM stage (III–IV vs. I–II)	2.247	1.041–4.852	0.039	2.018	1.062–3.835	0.032
Lymphovascular invasion (presence vs. absence)	3.180	1.160–8.714	0.025	3.402	1.433–8.079	0.006
Histopathological type (other vs. tubular adenocarcinoma)	3.290	1.019–10.630	0.047	2.769	1.042–7.355	0.041
Histopathological grade (poor vs. good and moderate)	2.526	1.028–6.206	0.043	1.693	0.818–3.503	0.156
hsa_circ_0001944	3.721	1.529–9.054	0.004	2.966	1.494–5.891	0.002

*HR* hazard ratios, *CI* confidence intervals

### Downregulation of hsa_circ_0001944 attenuated cell proliferation, invasion and migration but induced the cell apoptosis

**Fig. 2:**
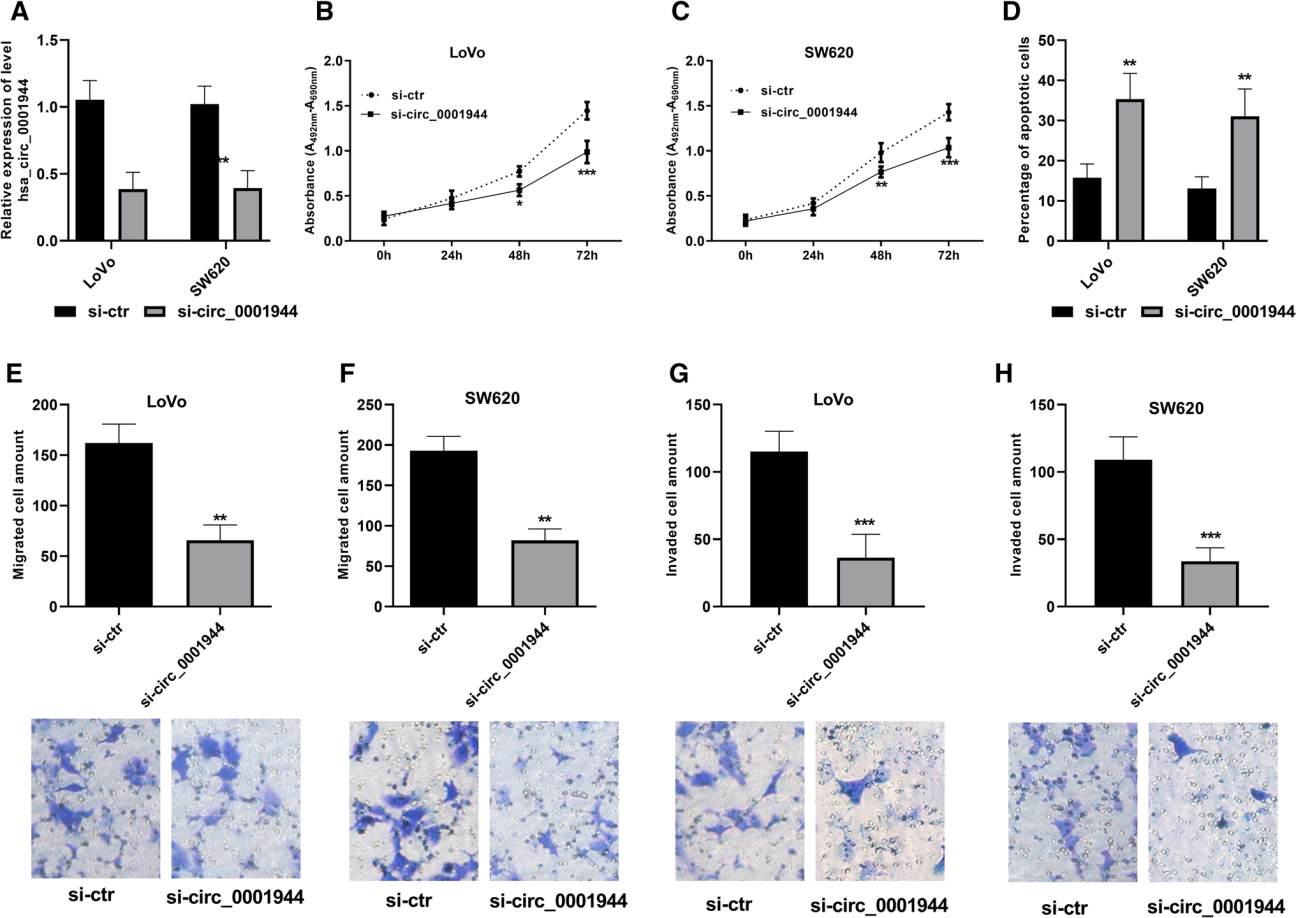
Inhibition of hsa_circ_0001944 decreased cell proliferation, reduced the migrated and invaded cells, but induced the cell apoptosis. **A** Verification of the transfection. **B**, **C** Cell proliferation was evaluated in LoVo and SW620 cells transfected with hsa_circ_0001944 siRNA (si-circ_0001944), using the negative siRNA as reference. **D** Quantitative statistical chart of the apoptotic cells. **E**, **F** Numbers of migrating cells were determined using Transwell assay. **G**, **H** Numbers of invading cells were determined using Matrigel-modified Transwell assay. Student t-test was applied. Error bars are mean ± standard error from three replicate samples. *P < 0.05, **P < 0.01***P < 0.001

To assess whether hsa_circ_0001944 has a role in CRC progression, siRNA targeting to hsa_circ_0001944 was used to decrease the upregulated expression of hsa_circ_0001944. Cell proliferation showed transfection of LoVo and SW620 cells with specific siRNAs reduced hsa_circ_0001944 levels by > 50%, as demonstrated by qRT-PCR (Fig. 2A and Supplementary Figure 1A). Cell proliferation assay showed that the effect of hsa_circ_0001944-specific siRNA decreased the cell growth, especially at the timepoint of 72 h (Fig. 2B, C; Supplementary Figure 1B, C). But the cell apoptosis was induced by inhibition of hsa_circ_0001944 (Fig. 2D). Cell migration and invasion were monitored by Transwell assay. LoVo and SW620 cells were transiently transfected with hsa_circ_0001944. Compared to control siRNA, we observed significantly attenuated migration in cells transfected with si-circ_0001944 (Fig. 2E, F; Supplementary Figure 1D). Similarly, robust inhibition of cell invasion was observed in hsa_circ_0001944 inhibited cells compared to negative ones (Fig. 2G, H; Supplementary Figure 1E).

### miR-548b-3p directly binds to hsa_circ_0001944

**Fig. 3:**
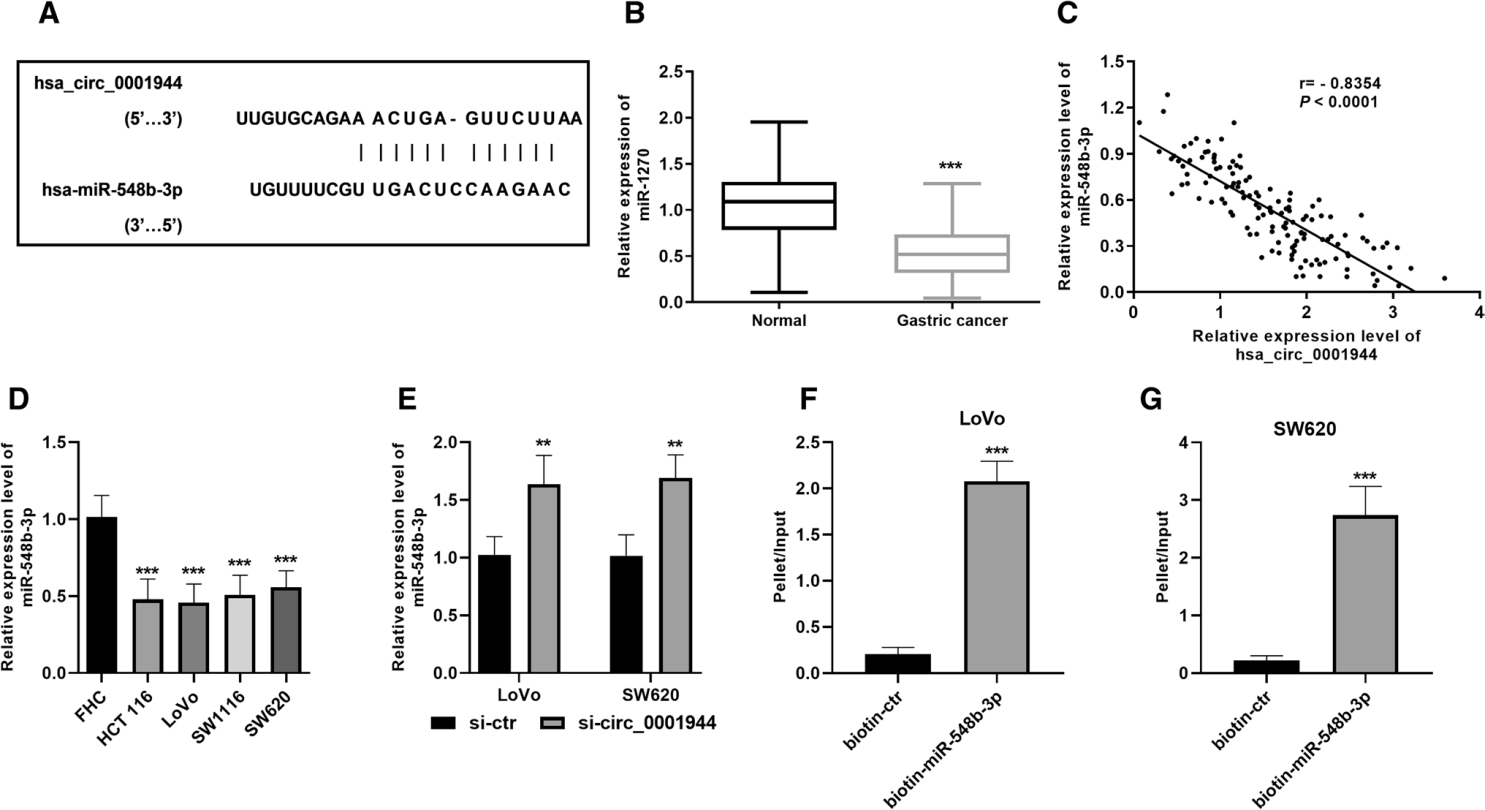
Hsa_circ_0001944 binds to miR-548b-3p. **A** The binding sites were predicted in Interactome. **B** miR-548b-3p was downregulated in CRC tissues. Paired t-test was applied. **C** The expression of hsa_circ_0001944 was negatively correlated with the expression of miR-548b-3p in CRC tissues (n = 133). Pearson correlation assay was applied. **D** miR-548b-3p was downregulated in CRC cells. **E** Inhibition of hsa_circ_0001944 increased the level of miR-548b-3p in CRC cells. Student t-test was applied. **F**, **G** RNA was affinity-isolated by biotinylated miR-548b-3p using biotinylated miRNA pull-down assay, and the hsa_circ_0001944 was quantified by qRT-PCR and normalized to input. Student *t*-test was applied. Error bars are mean ± standard error from at least three replicate samples. **P < 0.01, ***P < 0.001

The binding sites of hsa_circ_0001944 and miR-548b-3p are displayed in Fig. 3A, as revealed by retrieval from the bioinformatic database. The expression of miR-548b-3p in CRC tissues was significantly decreased than the normal tissues (Fig. 3B). The expression of hsa_circ_0001944 and miR-548b-3p in CRC tissues was negatively correlated (Fig. 3C). The expression level of miR-548b-3p in CRC cell lines confirmed the downregulation of miR-548b-3p in CRC (Fig. 3D). Further, the inhibition of hsa_circ_0001944 expression caused an increase of miR-548b-3p level in LoVo and SW620 cells (Fig. 3E). To confirm binding between hsa_circ_0001944 and miR-548b-3p, a biotin-coupled probe pull-down assay was performed and revealed that hsa_circ_0001944 was significantly enriched in the pulled down material compared to the control group, indicating that miR-548b-3p directly binds to hsa_circ_0001944 (Fig. 3F ,G).

### miR-548b-3p reversed hsa_circ_0001944-caused effect on cell proliferation, apoptosis, migration, and invasion in SW620 cells

**Fig. 4:**
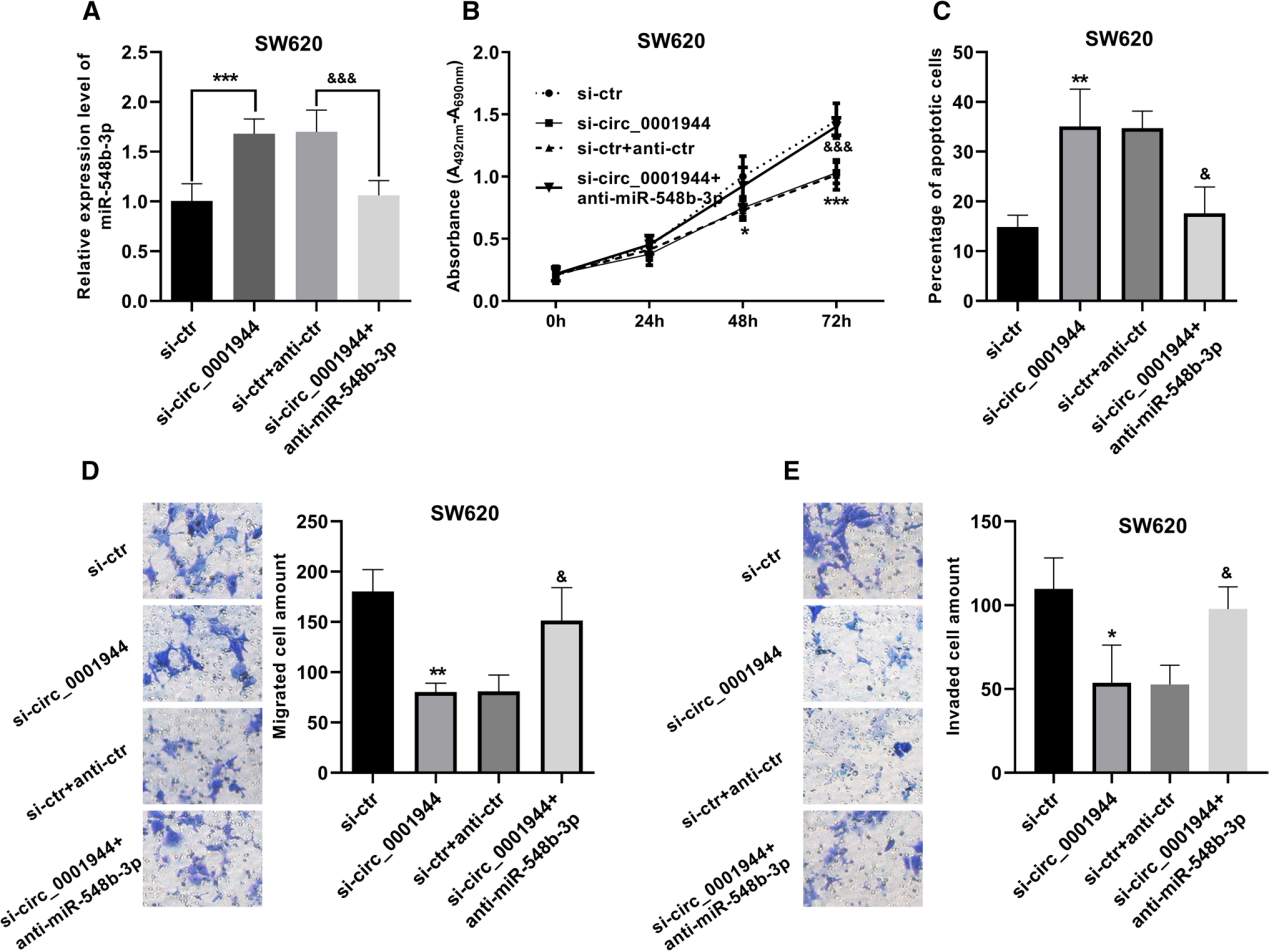
miRNA-548b-3p knockdown deteriorated hsa_circ_0001944-caused proliferation inhibition, migration inhibition, invasion inhibition, and apoptosis induction. **A** The transfection efficiency was determined by qRT-PCR. **B** Cell proliferation was evaluated by XTT assay. **C** Quantitative statistical chart of the apoptotic cells. **D**, **E** Numbers of migrating and invaded cells were determined using Transwell assay. *P < 0.05, **P < 0.01, ***P < 0.001 (vs si-ctr). ^&^*P* < 0.05, ^&&&^*P* < 0.001(vs si-ctr + anti-ctr)

To gain insight into the mechanism of hsa_circ_0001944 in CRC, we used SW620 cell line for rescue experiments. We moderated the expression level miR-548b-3p in the presence or absence of miR-548b-3p inhibitor (anti-miR-548b-3p) (Fig. 4A). Cell proliferation was detected when hsa_circ_0001944 was inhibited alone and co-inhibited with miR-548b-3p. Intriguingly, the suppression in cell proliferation caused by hsa_circ_0001944 siRNA was abolished when miR-548b-3p was additionally co-inhibited (Fig. 4B). Likewise, the cell apoptosis data displayed that miR-548b-3p inhibitor hindered hsa_circ_0001944-induced apoptosis (Fig. 4C). When hsa_circ_0001944 was co-inhibited with miR-548b-3p, the amount of migrated and invaded cells increased again (Fig. 4D, E). Taken together, these results demonstrate that miR-548b-3p could reverse hsa_circ_0001944-caused effect on cell proliferation, apoptosis, migration, and invasion.

## Discussion

Despite considerable advances in CRC therapies, improvements in the survival and prognosis of patients have not followed. A better understanding of CRC molecular biology and better characterization of newly discovered potential markers has important implications for CRC treatment and improvement of patient outcomes [20]. With the in-depth understanding of circRNAs, the potential applications provided by circRNAs continue to grow. As it becomes easy to collect circRNAs from patients through saliva and blood, circRNAs may one day be added to common clinical laboratory test libraries. Their ability as biomarkers can even be used as a screening tool for the prognosis of CRC. The ability to inhibit miRNA function and regulate protein function makes circRNAs not only the main gene regulator in the development and progress of CRC, but also a new therapeutic target with a more powerful effect in the treatment of CRC [21]. There is increasing evidence that circRNAs have the prognostic potential for CRC. Even using circRNAs alone can provide a more accurate prognostic prediction for CRC patients, so as to carry out treatment intervention earlier and more effectively. Aberrant circRNA expression between CRC and normal non-malignant cells or tissues is anticipated, and growing studies have supported this postulation. Moreover, it was found that the abnormal expression of circRNAs was closely related to CRC pathology [22]. The study of circRNA differentially expressed between healthy and cancer tissues would reveal the new role of circRNA as a potential target for CRC treatment.

hsa_circ_0001944 is located on chromosome Xq26.2 (1096 bp, chrX: 130883333–130928494) and derived from a long non-coding RNA region within the FIRRE locus. It has been verified to exist in a circular form and was mainly located in the cytoplasm [15]. Our study focused on defining a possible role for hsa_circ_0001944 as a potential OS-predictive and RFS-predictive biomarker in CRC. The expression of hsa_circ_0001944 in the current 133 pairs of tissues indicated the upregulation of hsa_circ_0001944 in CRC. This was consistent with the previous report that hsa_circ_0001944 was screened as an increased circRNA in CRC [16]. Based on the fact that dysregulation of hsa_circ_0001944 in CRC, we suppose hsa_circ_0001944 may be a potential marker for CRC prognosis. Interestingly, several studies have been reported to explore the predictive value of hsa_circ_0001944 in cancer prognosis: high hsa_circ_0001944 levels predicted unfavorable prognoses for bladder cancer, non-small cell lung cancer and breast cancer patients [17, 23, 24]. Prognostic and predictive biomarkers play a crucial role in cancer management because they can help clinicians choose the best treatment strategy for each patient. Our study suggests that up-regulation of hsa_circ_0001944 is associated with advanced cTNM stage and positive lymphovascular invasion. Further, hsa_circ_0001944 was confirmed to be a potent biomarker for CRC prognosis, both in OS and RFS. We, therefore, suggest that hsa_circ_0001944 is a promising candidate for predictive biomarkers in CRC prognosis.

Hsa_circ_0001944 plays a role in the regulation of multiple cellular processes. In bladder cancer, hsa_circ_0001944 promotes cell proliferation and invasion [18, 21]. Hsa_circ_0001944 can promote proliferation, migration, invasion, and glycolysis of lung cancer cells [19]. The results of in vitro cell culture model provide us with a promising potential of hsa_circ_0001944 in CRC. In this study, we demonstrated that high levels of hsa_circ_0001944 in CRC participate in the regulation of cell migration, invasion and growth. Hsa_circ_0001944 has been demonstrated as a tumor promoter in several cancers, and hsa_circ_0001944 up-regulation in CRC has previously been reported [16]. The up-regulated circRNAs in CRC were usually verified to promote cell growth and metastasis. Consequently, silencing the upregulated circRNAs effectively retards these processes. circHIPK3 is upregulated in CRC and promotes CRC through positively regulating the cell growth [25, 26]. Altogether, this evidence strongly suggests that hsa_circ_0001944 is onco-circRNA in CRC and potentially serves as a potential cellular target.

CircRNAs have been found to have many functions, ranging from acting as miRNA sponges, interacting with proteins to regulate transcription to allowing translation [27]. CircRNAs in different parts of cells has different biological functions. Those located in the cytoplasm mainly could act as miRNA sponges, while circRNAs dispersed in the nucleus have the effect of regulating the transcription of parental genes [28]. Understanding the mechanism of circRNAs function in CRC would provide valuable insight into the pathogenesis and potential in CRC therapeutics. The existing multiple direct binding sites for miRNAs make circRNAs play roles as miRNA sponges [29]. Therefore, the importance of these circRNAs in pathogenic pathways can be studied from the perspective of their target miRNAs. By sponging miRNA, circRNAs have the ability to reverse the silencing effect of their target miRNA and increase the expression of downstream genes. Has_circ_0001944 has been discovered to function through sponging miRNAs, for example, miR-142-5p in lung cancer and miR-20a-5p in acute myeloid leukemia [30]. In CRC, miR-548b-3p was found to be one of the target miRNAs for has_icrc_0001944 in this presented study. The expression of miR-548b-3p suppressed by has_circ_0001944 and the pull-down assay gave further support to the notion that has_circ_0001944 may promote CRC by sponging miR-548b-5p. This study demonstrated that miR-548b-3p could reverse hsa_circ_0001944-caused effect on CRC cell proliferation, apoptosis, migration, and invasion. miR-548b has been reported to suppress CRC through *WNT2* [31]. Altogether, it might be reasonable to conclude that has_circ_0001944 may act as a promoter in CRC by sponging miR-548b-5p and then releasing *WNT2*. This finding expands the understanding of the circRNA/miRNA regulatory network in CRC and suggests the therapeutic potential of silencing has_circ_0001944 in CRC.

In conclusion, the present retrospective study shows that the upregulation of has_circ_0001944 in CRC was associated with unfavorable prognosis, OS and RFS, for patients. This suggests that has_circ_0001944 may serve as promising prognosis-predictive biomarkers. Has_circ_0001944 may promote CRC by sponging miR-548b-5p. This suggests the therapeutic potential of silencing has_circ_0001944 in CRC.

## Supplementary Information

**Additional file 1: Figure S1.** si-circ_0001944-2 decreased cell proliferation and, reduced the migrated and invaded cells, but induced the cell apoptosis. (A) Verification of the transfection. (B) (C) Cell proliferation was evaluated in LoVo and SW620 cells transfected with si-circ_0001944-2, using the negative siRNA as reference. (D) Numbers of migrating cells were determined using Transwell assay. (E) Numbers of invading cells were determined using Matrigel-modified Transwell assay. *P < 0.05, **P < 0.01.

## Data Availability

All data generated or analysed during this study are included in this published article.
