# Supplementary figures and images for: Association of has_circ_0001944 upregulations with prognosis and cancer progression in patients with colorectal cancer
Source: Discov Oncol. 2022 Apr 9;13:23. doi: 10.1007/s12672-022-00485-2 (PMC8994801; doi:10.1007/s12672-022-00485-2)

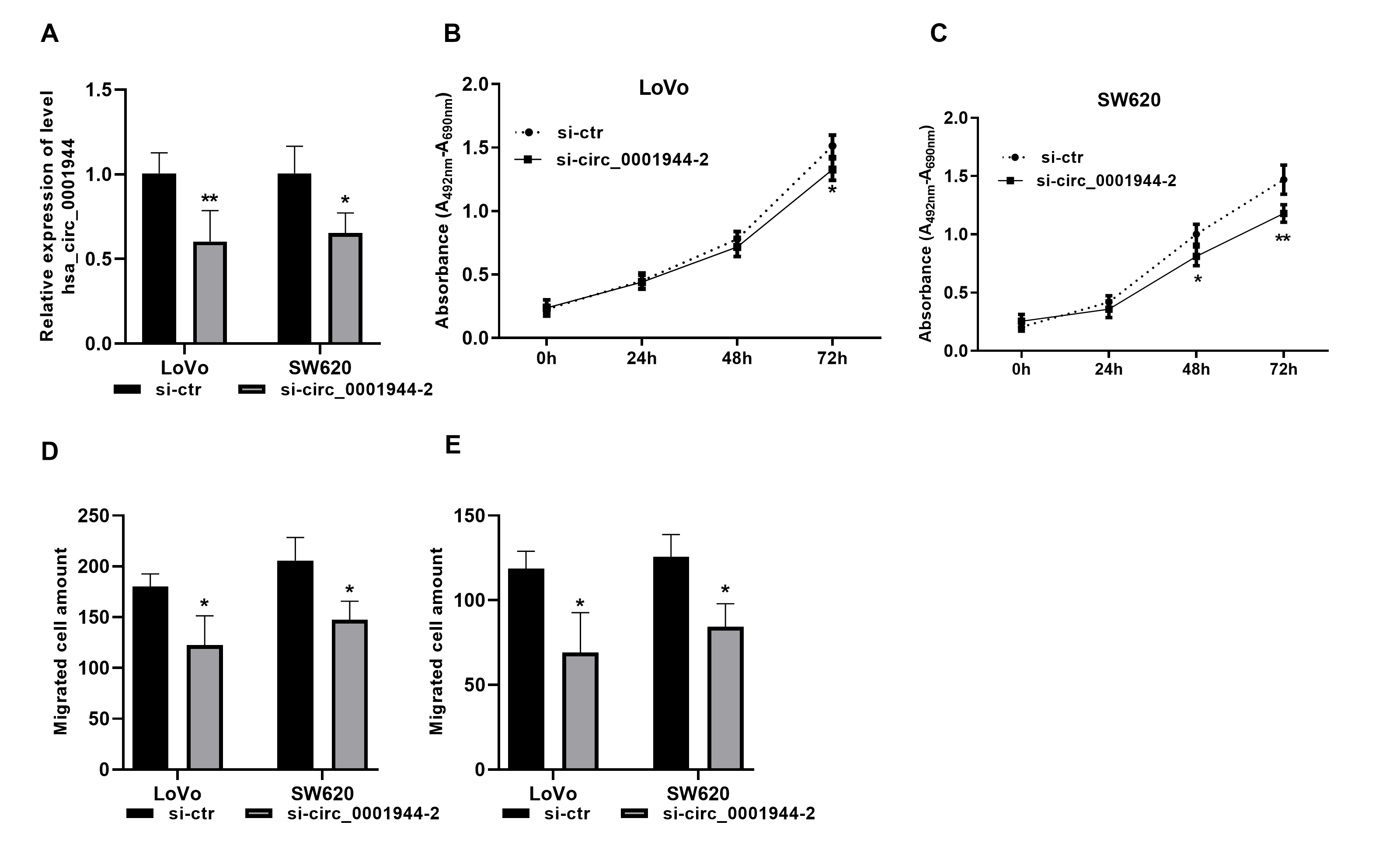

Supplement: Supplementary file 1 — Additional file 1: Figure S1. si-circ_0001944-2 decreased cell proliferation and, reduced the migrated and invaded cells, but induced the cell apoptosis. (A) Verification of the transfection. (B) (C) Cell proliferation was evaluated in LoVo and SW620 cells transfected with si-circ_0001944-2, using the negative siRNA as reference. (D) Numbers of migrating cells were determined using Transwell assay. (E) Numbers of invading cells were determined using Matrigel-modified Transwell assay. *P < 0.05, **P < 0.01. [file 12672_2022_485_MOESM1_ESM.tif]
